# Prognostic value of MRI‐determined cervical lymph node size in nasopharyngeal carcinoma

**DOI:** 10.1002/cam4.3392

**Published:** 2020-08-13

**Authors:** Cheng‐Long Huang, Yang Chen, Rui Guo, Yan‐Ping Mao, Cheng Xu, Li Tian, Li‐Zhi Liu, Ai‐Hua Lin, Ying Sun, Jun Ma, Ling‐long Tang

**Affiliations:** ^1^ Department of Radiation Oncology State Key Laboratory of Oncology in South China Collaborative Innovation Center for Cancer Medicine Guangdong Key Laboratory of Nasopharyngeal Carcinoma Diagnosis and Therapy Sun Yat‐sen University Cancer Center Guangzhou China; ^2^ Imaging Diagnosis and Interventional Center State Key Laboratory of Oncology in South China Collaborative Innovation Center for Cancer Medicine Guangdong Key Laboratory of Nasopharyngeal Carcinoma Diagnosis and Therapy Sun Yat‐sen University Cancer Center Guangzhou China; ^3^ Department of Medical Statistics and Epidemiology School of Public Health Sun Yat‐sen University Guangzhou China

**Keywords:** lymph node, magnetic resonance, N staging system, nasopharyngeal carcinoma, prognosis

## Abstract

**Objectives:**

To investigate the prognostic value of magnetic resonance imaging (MRI)‐determined cervical lymph node (CLN) size in nasopharyngeal carcinoma (NPC).

**Methods:**

We retrospectively reviewed 2066 patients with NPC treated with intensity‐modulated radiotherapy, and randomly divided them into two groups, in a 1:1 ratio. One group was used for training (the training group), and the other one was for internal validation (the validation group). All patients had undergone MRI examination and the maximal axial diameters (MAD) of the axial plane of all positive nodes had been measured and recorded.

**Results:**

Of 683 patients with CLN metastases in the training group (n = 1033), MAD = 4 cm was associated with worse OS (64.7% vs 84.6%, *P* < .001), DFS (55.9% vs 76.3%, *P* = .001), and DMFS (67.6% vs 86.1%, *P* = .001). Multivariate analysis showed that MAD = 4 cm was a significant negative prognostic factor for OS (HR = 2.058; *P* = .025), DFS (HR = 1.727; *P* = .049), and DMFS (HR = 2.034; *P* = .036). When MRI‐determined MAD = 4 cm was classified as N3 in the N classification, the OS, DFS, DMFS, and RRFS survival curves were well separated. The OS, DFS, DMFS, and RRFS concordance indexes were not statistically different between the proposed N staging system and the UICC/AJCC staging system in the training group, or between the training group and the validation group (all *P* = .05).

**Conclusion:**

MAD = 4 cm on axial MRI slices can be recommended as a prognostic factor in future versions of the UICC/AJCC NPC staging system.

## Graphical abstract

We identified the cut‐off value of the greatest diameter of lymph nodes on axial MRI slices, that is, MAD > 4 cm. MAD = 4 cm was associated with worse survival outcome in nasopharyngeal carcinoma. MAD == 4 cm could be recommended as a prognostic factor in future versions of the UICC/AJCC staging system.

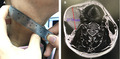

## 1 INTRODUCTION

Nasopharyngeal carcinoma (NPC) is endemic in Southern China. On account of the rich nasopharyngeal lymphatic network, lymph node (LN) metastasis is common in NPC.^1^ About 75% of pretreatment patients have enlarged neck node(s).^2^ Palpation‐determined LN size (greatest diameter ≤6 vs >6 cm) was found to be a significant prognostic factor for NPC,^3^ and consequently it was included in the Union for International Cancer Control/American Joint Committee on Cancer (UICC/AJCC) NPC staging system N3 subset in 1992 (ie the 4th edition). Subsequent staging systems have also adopted this criterion, including the 8th edition of the UICC/AJCC staging system. However, the CLN criterion is based on the evaluation of palpable LNs, which would be influenced by subcutaneous tissue and might differ among clinicians. Furthermore, the wide use of computed tomography (CT), magnetic resonance imaging (MRI), positron emission tomography (PET)‐CT, and other advanced techniques in NPC detects LN metastases at a higher rate of 77%–88.1%^4^, ^5^ compared to only about 60% by palpation;^6^ MRI‐determined LN size would be more accurate than that determined by palpation. The MRI‐determined longest CLN diameter is a significant prognostic factor of both disease failure and distant failure in NPC.^7^ Hence, there is a need to investigate the cut‐off value of MRI‐determined LN size in the staging system.

Here, we investigated the significance of MRI‐determined nodal size on NPC treatment outcome and its value for the N staging system, which would meet the requirements of diagnostic development and reduce the subjective difference between centers or clinicians.

## 2 MATERIALS AND METHODS

### 2.1 Patients

We retrospectively reviewed 2066 patients with newly diagnosed, nondistant metastatic, histologically proven NPC treated at our cancer center between January 2010 and June 2012. All patients underwent a pretreatment evaluation, including complete patient history, physical examination, hematology and biochemistry profiles, MRI of the nasopharyngeal and neck, chest radiography, abdominal sonography, and single‐photon emission CT whole‐body bone scan. Additionally, 571 patients (27.6%) underwent ^18^F‐fluorodeoxyglucose PET/CT examination. The clinical research committee of the study institute approved the study protocol, and written informed consent was waived by the Institutional Review Board. All patients were restaged according to the 8th edition of the UICC/AJCC system. Table 1 summarizes the patients’ clinical characteristics.

**Table 1:** Clinical characteristics of the 2066 patients with NPC

Characteristic	Patients number
Gender	
Male	1545 (74.8%)
Female	521 (25.2%)
Age (y)	
<45	1002 (48.5%)
≥45	1064 (51.5%)
Histological type	
Keratinizing squamous cell carcinoma	11 (0.5%)
Nonkeratinizing carcinoma	2055 (99.5%)
Chemotherapy	
No	271 (13.1%)
Yes	1795 (86.9%)
Induction chemotherapy	1013(49.0%)
Concurrent chemotherapy	1541(74.6%)
Adjuvant chemotherapy	65(3.1%)
T category	
T1	363 (17.6%)
T2	331 (16.0%)
T3	992 (48.0%)
T4	380 (18.4%)
N category	
N0	698 (33.8%)
N1	796 (38.5%)
N2	303 (14.7%)
N3	269 (13.0%)
Overall stage	
I	176 (8.5%)
II	351(17.0%)
III	938 (45.4%)
IV	601 (29.1%)

Abbreviation: NPC, nasopharyngeal carcinoma.

### 2.2 Imaging protocol and image assessment

**Figure 1:**
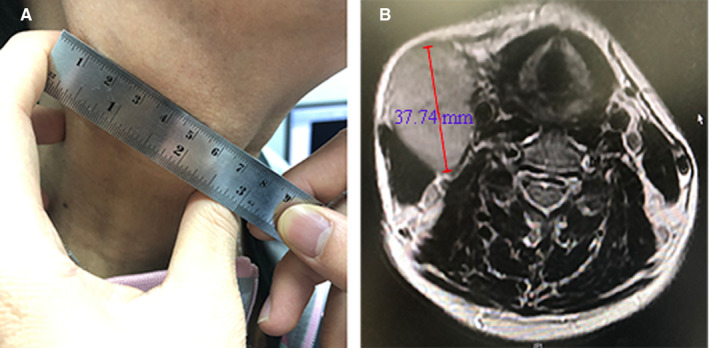
An example of different measurement methods and results of the same lymph node. A patient aged between 40 and 60 y old was diagnosed as N3 NPC, with the greatest diameter of the cervical lymph node = 6.50 cm by palpation (A); while in MRI, the maximal axial diameter of the same node = 37.74 mm (B)

The imaging protocol, the diagnostic criteria for LN metastases, and the assessment of LN location were described previously.^8^ Two masked radiologists specializing in head and neck cancers evaluated all scans separately. Any disagreements were resolved by consensus. The axial plane maximal axial diameter (MAD) of all positive nodes was measured and recorded (Figure 1). If two nodes had fused, those could still be distinguished from each other were measured individually; otherwise, the diameter of the confluent nodes was recorded. After assessment of all the LNs, the two radiologists commonly determined which LN was the largest when one patient had several positive LNs. The value of MAD used for analysis in the present study was the average of the results measured by the two radiologists. The median variance was 0.123 (range, 0.006, 5.06).

### 2.3 Treatment

All patients were treated with intensity‐modulated radiotherapy (IMRT). Target volumes (TVs) were delineated according to the International Commission on Radiation Units and Measurements reports 50 and 62. The prescribed RT doses were described previously.^9^ According to our institutional guidelines, concurrent chemoradiotherapy ± neoadjuvant/adjuvant chemotherapy was recommended for stage II–IVB NPC; patients with N2‐3 disease, T4 disease, or with relatively large gross tumor volume were more likely to receive neoadjuvant chemotherapy; and patients with detectable plasma EBV DNA after treatment or with residual tumor were more likely to receive adjuvant chemotherapy. When possible, patients with persistent or progressive disease received salvage treatments (reirradiation, surgery, or chemotherapy).^7^

### 2.4 Endpoints and follow‐up

The following endpoints were assessed: overall survival (OS), disease‐free survival (DFS), distant metastasis–free survival (DMFS), and regional relapse–free survival (RRFS). Beginning from treatment day 1, OS was defined as the time to the date of death from any cause or last follow‐up, whichever occurred first; DFS, the time to failure, death, or last follow‐up; DMFS, the time to distant metastasis or last follow‐up; and RRFS, the time to nodal relapse or last follow‐up. Patients with suspected lesions underwent fine‐needle aspiration or biopsy to confirm malignancy, if necessary.

Patients were assessed every 3 months during the first 2 years of follow‐up, then every 6 months for at least 3 years, and annually thereafter until death. The median follow‐up time was 60.2 months (range 1.2‐83.1 months). The last follow‐up time was October 10, 2018.

### 2.5 Statistical analysis

Statistical analyses were performed using SPSS version 24.0 (IBM). We randomly divided the whole patients into two groups (the training group and the validation group), in a 1:1 ratio. The training group was used to investigate the cut‐off value of MRI‐determined LN size and to propose a new N3 subset, and the validation group was used for internal validation. Survival outcomes were analyzed using the Kaplan‐Meier method, and survival curves were compared using the log‐rank test.^10^ Stratified Cox proportional hazards model was used to test for independent significance by backward elimination of insignificant explanatory variables^11^ and to calculate hazard ratios (HR). Age, gender, and chemotherapy history were included as covariates in all tests. The performance of the UICC/AJCC staging system and the proposed staging system were compared using Harrell's concordance index (c‐index),^12^ which measures the ability to predict outcomes: a higher c‐index suggests a greater ability to discriminate outcomes (ie the model has better discriminatory power). *P* < .05 was considered statistically significant.

## 3 RESULTS

### 3.1 Characteristics of nodal spread

The incidence of LN metastases, retropharyngeal lymph nodes (RLN), and CLN metastases was 83.0% (1715/2066), 75.2% (1553/2066), and 66.2% (1368/2066), respectively. The incidence of palpation‐determined CLN metastases was 56.3% (1163/2066), which was significantly different from the incidence of MRI‐determined CLN metastases (66.2%, 1368/2066, *P* < .001).

All 1368 patients with positive CLNs were analyzed. Bilateral cervical node involvement was observed in 428 patients (31.3%). The mean MRI‐determined axial plane MAD of the positive CLNs was 2.4 ± 0.9 cm (range, 0.9‐8.1 cm). The categorization of MAD was as follows: MAD ≤ 2 cm; 2 cm < MAD ≤ 3 cm; 3 cm < MAD ≤ 4 cm; and MAD = 4 cm. Since only 21 patients (1.5%) had nodal MAD = 5 cm, and 6 patients (0.4%) had MAD = 6 cm among those with node metastasis, we did not subgroup the nodes with MAD = 4 cm further. Nodal size correlated statistically with level (above vs below level IV and Vb) and laterality (bilateral vs unilateral) (both, *P* < .001, Table S1).

### 3.2 Prognostic value of MRI‐determined CLN MAD

In the training group (n = 1033), 683 patients had positive CLNs. Univariate analysis showed that MAD = 4 cm was associated with significantly worse OS (64.7% vs 84.6%, *P* < .001), DFS (55.9% vs 76.3%, *P* = .001), and DMFS (67.6% vs 86.1%, *P* = .001), but not with RRFS (91.2% vs 93.8%, *P* = .340); MAD = 3 cm was associated with significantly worse OS (77.0% vs 85.4%, *P* = .009) and RRFS (90.5% vs 94.6%, *P* = .043), but not with DFS (70.3% vs 76.6%, *P* = .064) or DMFS (81.8% vs 86.2%, *P* = .139); and MAD = 2 cm was not associated with worse OS (82.2% vs 86.3%, *P* = .169), DFS (73.6% vs 78.3%, *P* = .136), DMFS (83.3% vs 88.8%, *P* = .054), or RRFS (92.6% vs 95.8%, *P* = .081) (Table 2).

**Table 2:** Clinical outcome of patient subsets segregated by MAD

Variables	OS	DFS	DMFS	RRFS
MAD value	*P* value	*P* value	*P* value	*P* value
	HR (95% CI)	HR (95% CI)	HR (95% CI)	HR (95% CI)
>4 vs ≤4 cm	***P* < .001**	***P* = .001**	***P* = .001**	*P* = .340
	HR = 2.83 (1.55‐5.14)	HR = 2.35 (1.38‐3.99)	HR = 2.82 (1.51‐5.27)	HR = 1.76 (0.54‐5.68)
>3 vs ≤3 cm	***P* = .009**	*P* = .064	*P* = .139	***P* = .043**
	HR = 1.70 (1.13‐2.54)	HR = 1.38 (0.98‐1.95)	HR = 1.39 (0.90‐2.16)	HR = 1.91 (1.01‐3.62)
>2 vs ≤2 cm	*P* = .169	*P* = .136	*P* = .054	*P* = .081
	HR = 1.33 (0.89‐1.99)	HR = 1.28 (0.92‐1.78)	HR = 1.54 (0.99‐2.39)	HR = 1.86 (0.92‐3.78)

Note

Clinical outcome was described by Kaplan‐Meier plots of OS, DFS, DMFS, and RRFS *P* < 0.05 are highlighted in bold.

Abbreviations: CI, confidence interval; DFS, disease‐free survival; DMFS, distant metastasis–free survival; HR, hazard ratio; MAD, maximal axial diameter; OS, overall survival; RRFS, regional relapse–free survival.

As there was high correlation between MAD = 2 cm (no vs yes), MAD > 3 cm (no vs yes), and MAD = 4 cm (no vs yes), the multivariate models only included one dosimetric parameter at one time during analyses to avoid multicollinearity. Age (<45 vs ≥45 years), gender, T classification, chemotherapy, CLN laterality (unilateral vs bilateral), and CLN level (above vs below the caudal border of the cricoid cartilage) were included in all multivariate analyses. Bilateral CLN and CLN level (below the caudal border of the cricoid cartilage) was independent negative prognostic factors of OS, DFS, and DMFS (all, *P* < .05). MAD = 4 cm was an independent negative prognostic factor of OS (HR = 2.058; *P* = .025), DFS (HR = 1.727; *P* = .049), and DMFS (HR = 2.034; *P* = .036), but not for RRFS (HR = 1.354; *P* = .620) (Table 3). MAD = 3 cm was an independent negative prognostic factor only for RRFS (HR = 1.912, *P* = .046), and MAD = 2 cm was not an independent prognostic factor for any endpoint.

**Table 3:** Summary of multivariate analysis of prognostic factors in 683 patients with NPC and CLN metastases

Endpoint	Variable	HR	95% CI for HR	*P‐*value*
OS (112 events)	Age, y (≥45 vs <45)	1.616	1.090‐2.395	**.017**
	T classification	1.368	1.100‐1.701	**.005**
	CLN level	2.605	1.746‐3.886	**<.001**
	CLN laterality	2.000	1.365‐2.931	**<.001**
	MAD	2.058	1095‐3.869	**.025**
DFS (169 events)	T classification	1.336	1.121‐1.592	**.001**
	CLN level	2.202	1.559‐3.110	**<.001**
	CLN laterality	1.579	1.156‐2.159	**.004**
	MAD	1.727	1.002‐2.976	**.049**
	Chemotherapy	0.517	0.288‐0.925	**.026**
DMFS (101 events)	T classification	1.434	1.140‐1.802	**.002**
	CLN level	2.982	1.969‐4.518	**<.001**
	CLN laterality	1.783	1.193‐2.666	**.005**
	MAD	2.034	1.047‐3.951	**.036**
RRFS (43 events)	CLN laterality	1.804	0.988‐3.295	.055

Abbreviations: CI, confidence interval; CLN, cervical lymph node; DFS, disease‐free survival; DMFS, distant metastasis–free survival; HR, hazard ratio; MAD, maximal axial diameter; NPC, nasopharyngeal carcinoma; OS, overall survival; RRFS, regional relapse–free survival.

* *P‐*values were calculated using an adjusted Cox proportional hazards model; the following known important prognostic variables were included: age (≥45 vs <45 y), gender (female vs male), T classification, CLN laterality (unilateral vs bilateral), CLN level (above vs below the caudal border of the cricoid cartilage), chemotherapy (yes vs no), and MAD values (≤4 vs >4 cm). *P* < 0.05 are highlighted in bold.

### 3.3 MRI‐determined CLN MAD in the staging system

As mentioned above, the 8th edition of the UICC/AJCC staging system adopted the greatest diameter >6 cm as a N3 subset according to the results from palpation. In this study with a relatively large sample size, the number of patients with nodal MAD = 5 cm or >6 cm was rather small that adopting the cut‐off value of 6cm for MAD might be not feasible. We proposed MAD = 4 cm as one of the N3 subsets in the N classification.

**Figure 2:**
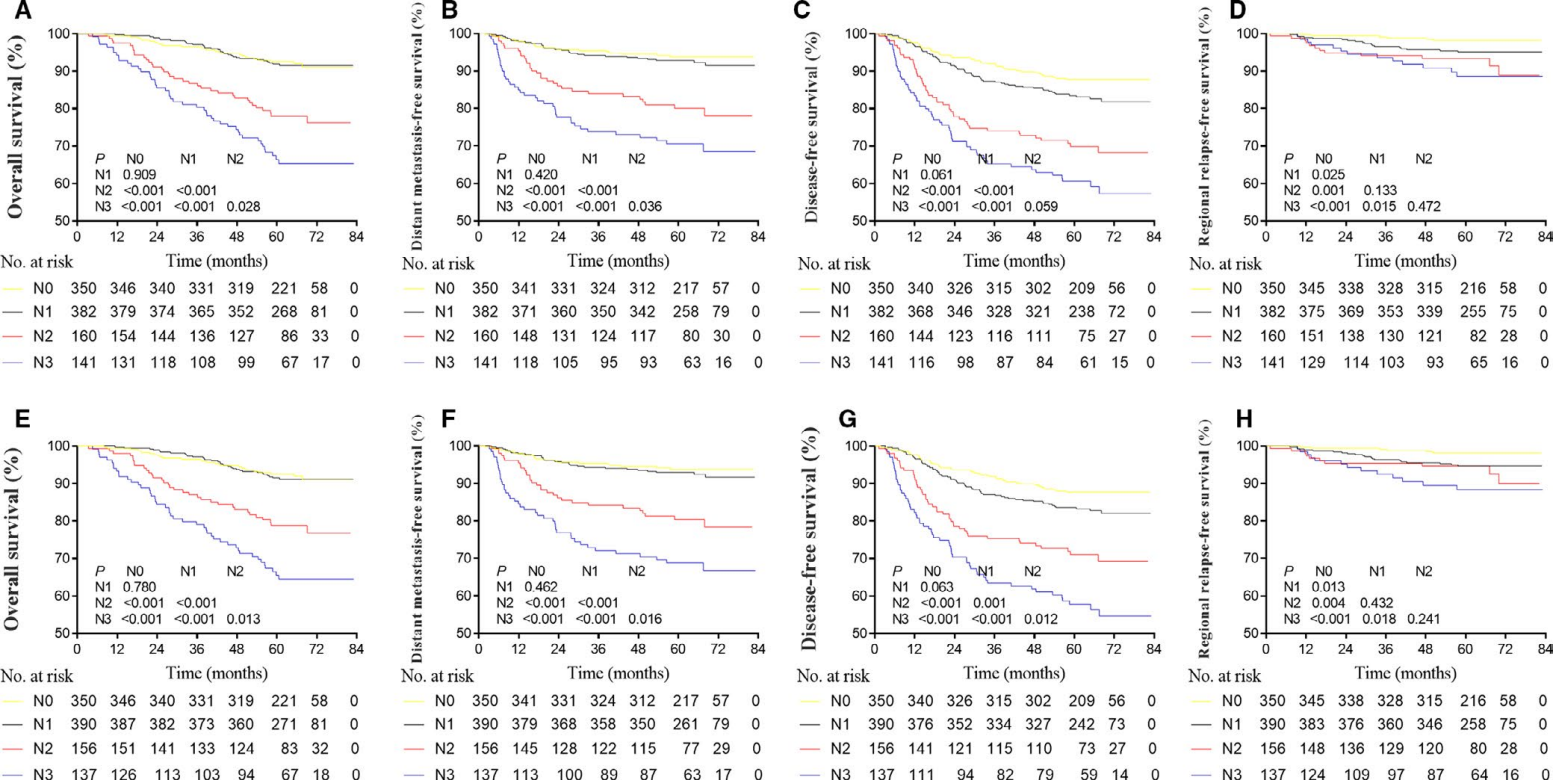
The OS, DFS, DMFS, and RRFS survival curves in the proposed N staging system when MAD = 4 cm was classified as N3, and in the UICC/AJCC staging system (8th edition). OS, overall survival; DFS, disease‐free survival; DMFS, distant metastasis–free survival; RRFS, regional relapse–free survival; MAD, maximal axial diameter

When MRI‐determined MAD = 4 cm was classified as N3 in the proposed N classification, 8 (1.2%) patients of the 683 patients with LN metastasis were upgraded from N1‐2 to N3, and 12 (1.8%) patients were downgraded from N3 to N1‐2 (Table S2). The OS, DFS, DMFS, and RRFS survival curves were separated both in the proposed N staging system and the UICC/AJCC staging system (Figure 2). The OS, DFS, DMFS, and RRFS c‐indexes of the proposed N staging system were higher than that of the UICC/AJCC staging system, although the difference was not statistically significant (all *P* = .05, Table S3).

### 3.4 Internal validation

The OS, DFS, DMFS, and RRFS c‐indexes of the proposed N staging system were not significantly different between the training and the validation group (all *P* = .05, Table S3).

## 4 DISCUSSION

In the present study, we investigate the prognostic value of MRI‐determined CLN size in NPC and found MAD of the positive nodes >4 cm was an independent negative prognostic factor for NPC. Furthermore, when we proposed MAD = 4 cm rather than MAD = 6 cm as an N3 subset criterion in the staging system, the survival curves were well separated.

In the 8th edition of the UICC/AJCC staging system, the lower neck, which was defined by imaging, replaced the supraclavicular fossa based on palpation and was included as an N3 subset criterion. This made demarcation easier and more reproducible. However, another N3 subset criterion, greatest diameter >6 cm based on the evidence from palpation, is not completely satisfactory. First, measurement could be interfered by the superficial structure including skin, subcutaneous fat, and neck muscles, which may differ among clinicians. The MAD on axial MRI slices is usually smaller than palpation‐based measures; only 0.2% and 0.7% of patients had MAD = 6 cm or MAD = 5 cm, respectively.^8^ Second, palpation cannot easily distinguish the fusion of multiple nodes. Rather than palpation, MRI could be more accurate in evaluation of LN. Lastly, criterion based on palpation is hard to determine on cross‐sectional imaging, and could not be universally described when radiologists interpreted imaging data sets. Therefore, there is a need to investigate the prognostic value and staging classification of MRI‐determined CLN size.

The UICC/AJCC N category defines NPC nodal size according to the largest dimension without specifying the plane to use. In the present study, we used the axial plane MAD as the index of MRI‐determined CLN size, but not the longest diameter of all planes defined in the UICC/AJCC tumor‐node‐metastasis system (eg coronal or sagittal planes). There are several reasons for this. First, newly diagnosed NPC without distant metastasis is typically treated with nonsurgical intervention. An unfavorable effect of CLN biopsy on survival outcomes has been observed in NPC patients,^13^ hence, CLN metastases are routinely diagnosed by radiologic criteria rather than histopathology. The cross‐section size is considered the most useful radiologic criteria for assessing CLN metastasis.^14^ Second, individual nodes and aggregated nodes are assessed more clearly on the axial plane, and we attempted to measure the individual nodes but not entire masses of aggregated nodes unless the nodes had truly fused and could not be distinguished from each other, according to the Response Evaluation Criteria in Solid Tumors guidelines.^15^ Lastly, MAD is easy to be measure in CT and MRI data sets or TV delineation of radiotherapy.

There are other LN parameters associated with prognosis in NPC, including gross nodal volume.^16^, ^17^ LN size (MAD) could be reasonably considered the surrogate for gross nodal volume. LN size is statistically correlated with other variables, including level and laterality, which have been included into the staging system as criteria. In multivariate analysis including level and laterality, we found CLN size (MAD = 4) was still an independent adverse prognostic factor for NPC, which was different from the result in Li's study^8^ that MAD with the cut‐off value of 3cm remained no longer the prognosticator for survival in multivariate analysis. One possible reason was the relatively small sample size in that study. Extracapsular spread (ECS) is also a negative prognostic factor in NPC.^18^ However, the identification of ECS in NPC is based on imaging rather than pathology and therefore is more subjective than the identification of other features of malignancy and results in wider variation in interpretation between centers, so CLN size is a more suitable factor than ECS in N classification.

The main limitation of this study was that the cases were all from a single center. Therefore, the applicability of these findings should be validated by data from external centers further. Moreover, the results from this retrospective study should be confirmed in a prospective study.

In conclusion, in the era of IMRT, MAD = 4 cm on axial MRI slices is recommended in future versions of the UICC/AJCC N staging system for NPC.

## CONFLICT OF INTEREST

The authors declare that they have no competing financial interests.

## AUTHOR CONTRIBUTIONS

CLH, JM, and LLT conceived, designed, and supervised the project. CLH, YC, RG, YPM, CX, LT, LZL, and LLT contributed to the design of the study, writing the protocol, data preparation, analysis, and interpretation. CLH and LLT drafted the manuscript. CX, AHL, YS, JM, and LLT performed the quality assessment and revised the manuscript. All authors have read and approved the submitted version.

## Supporting information

Table S1

Table S2

Table S3

## Data Availability

The data that support the findings of this study are available from the corresponding author upon reasonable request.
