# Supplementary material for: Prognostic value of MRI‐determined cervical lymph node size in nasopharyngeal carcinoma
Source: Cancer Med. 2020 Aug 13;9(19):7100–6. doi: 10.1002/cam4.3392 (PMC7541162; doi:10.1002/cam4.3392)
Supplement: Supplementary file 1 — Table S1 [file CAM4-9-7100-s001.docx]

**Supplementary Table S1.** Correlation between LN size and other nodal parameters.

| Parameter | LN size | | | | *P*-value | |
| --- | --- | --- | --- | --- | --- | --- |
|  | MAD ≤2cm | 2< MAD ≤3cm | 3< MAD ≤4cm | MAD >4cm |  |  |
| Level IV, Vb |  |  |  |  |  |  |
| (-) | 466(92.6%*) | 474(83.5%) | 156(69.0%) | 40(56.3%) | ＜0.001 |  |
| (+) | 37(7.4%) | 94 (16.5%) | 70(31.0%) | 31(43.7%) |  |  |
| Laterality |  |  |  |  |  |  |
| Unilateral | 415(82.5%*) | 362(63.7%) | 131(58.0%) | 32(45.1%) | ＜0.001 |  |
| Bilateral | 88(17.5%) | 206(36.3%) | 95(42.0%) | 39(54.9%) |  |  |

Abbreviations: LN, lymph node; MAD, maximal axial diameter.

* column %. (-): above the caudal border of the cricoid cartilage; (+): below the caudal border

of the cricoid cartilage.
