# Supplementary material for: Prognostic value of MRI‐determined cervical lymph node size in nasopharyngeal carcinoma
Source: Cancer Med. 2020 Aug 13;9(19):7100–6. doi: 10.1002/cam4.3392 (PMC7541162; doi:10.1002/cam4.3392)
Supplement: Supplementary file 2 — Table S2 [file CAM4-9-7100-s002.docx]

**Supplementary Table S2.** The distribution of the 8^th^ edition of the UICC/AJCC N staging system and the proposed N staging system

| UICC/AJCC staging system | Proposed staging system | | | | Total |
| --- | --- | --- | --- | --- | --- |
|  | 0 | 1 | 2 | 3 |  |
| 0 | 350(33.9%) | 0 | 0 | 0 | 350(33.9%) |
| 1 | 0 | 380(36.8%) | 0 | 2(0.2%) | 382(37.0%) |
| 2 | 0 | 0 | 154(14.9%) | 6(0.6%) | 160(15.5%) |
| 3 | 0 | 10(1.0%) | 2(0.2%) | 129(12.4%) | 141(13.6%) |
| Total | 350(33.9%) | 390(37.8%) | 156(15.1%) | 137(13.2%) | 1033(100.0%) |

Data are the number of patients; percentages are in parentheses.
