# Supplementary material for: Prognostic value of MRI‐determined cervical lymph node size in nasopharyngeal carcinoma
Source: Cancer Med. 2020 Aug 13;9(19):7100–6. doi: 10.1002/cam4.3392 (PMC7541162; doi:10.1002/cam4.3392)
Supplement: Supplementary file 3 — Table S3 [file CAM4-9-7100-s003.docx]

**Supplementary Table S3.** The OS, DFS, DMFS, and RRFS c-indexes of the proposed N staging system and the 8^th^ edition of the UICC/AJCC N staging system.

| Item |  | OS | DFS | DMFS | RRFS |
| --- | --- | --- | --- | --- | --- |
|  |  | C-index (95% CI)  *P* value | C-index (95% CI)  *P* value | C-index (95% CI)  *P* value | C-index (95% CI)  *P* value |
| Proposed N staging system (training group) |  | 0.693 (0.646,0.739)  *P* (reference) | 0.650 (0.611,0.690)  *P* (reference) | 0.687 (0.633,0.740)  *P* (reference) | 0.665 (0.594,0.735)  *P* (reference) |
| 8th N staging system (training group) |  | 0.692 (0.646,0.739)  *P* =0.987 | 0.649 (0.610,0.688)  *P* =0.956 | 0.683 (0.629,0.737)  *P* =0.928 | 0.673 (0.604,0.742)  *P* =0.868 |
| Proposed N staging system (validation group) |  | 0.653 (0.608,0.699)  *P* =0.237 | 0.637 (0.599,0.675)  *P* =0.644 | 0.680 (0.633,0.728)  *P* =0.869 | 0.698 (0.636,0.761)  *P* =0.484 |

Abbreviations: OS, overall survival; DFS, disease-free survival; DMFS, distant metastasis-free survival; RRFS, regional relapse-free survival;

CI, confidence interval.
